# Cheap Ice Houses

**Published:** 1874-09

**Authors:** 


### CHEAP ICE HOUSES.

Set up posts in the ground two feet apart, and closely board them on the inside, so as to make an enclosure ten feet each way in the clear, with a loose flooring, have a steep roof, with a door in it on the north side.

Pack in the ice in blocks as close together as possible, with an interval of half a foot or more between the ice and the sides of the structure; fill this space with cut straw, saw dust, or tan, and ram it down well, with six inches depth of the same on the floor and a foot or more on the top. Structure and packing need not cost forty dollars. The floor or ground should be on an incline, so as to carry off any water rapidly. It is the air coming between the pieces of ice which causes it to melt rapidly, hence if water could be slowly introduced on an intensely cold night, the whole would be a solid block by the morning, and would last two years for one ordinary family. In some cases the crevices might be filled with pounded ice, on a very cold day, or with spare tan or cut straw. Take out the ice as quickly as possible, cover up the place, and shut the door.

If ice has to be brought from a distance for the sick, it will keep a long time between two feather pillows; or if put in a stone jar, stand this on a pillow, lay another pillow around the jar and cover it with another, and keep the whole in a shady place.

